# Complete Genome Sequence of Heat-Resistant Newcastle Disease Virus Strain HR09

**DOI:** 10.1128/genomeA.01149-17

**Published:** 2017-11-02

**Authors:** Yongzhong Cao, Qian Liu, Xiaorong Zhang, Hai Hu, Yantao Wu

**Affiliations:** a Institutes of Agricultural Science and Technology Development, Yangzhou University, Yangzhou, China; b Joint International Research Laboratory of Agriculture and Agri-Product Safety, Yangzhou University, Yangzhou, China; c College of Veterinary Medicine, Yangzhou University, Yangzhou, China; d Jiangsu Co-innovation Center for Prevention and Control of Important Animal Infectious Diseases and Zoonoses, Yangzhou, China

## Abstract

In this study, the complete genome sequence of the heat-resistant Newcastle disease virus (NDV) strain HR09, which was isolated in China in the 1990s, was determined and characterized phylogenetically. This is the first report regarding the complete genomic information of a thermostable NDV strain from China.

## GENOME ANNOUNCEMENT

Newcastle disease (ND) is a contagious disease of poultry. Its causative pathogen, ND virus (NDV) (*Avian avulavirus 1*), which belongs to the family *Paramyxoviridae*, is an enveloped virus with a nonsegmented, single-stranded, and negative-sense RNA genome. The NDV genome contains six genes encoding the nucleoprotein (NP), phosphoprotein (P), matrix protein (M), fusion protein (F), hemagglutinin-neuraminidase protein (HN), and large polymerase protein (L). NDV can be divided into two classes, which include at least 9 or 10 genotypes based on F gene sequence analysis (1–3).

According to their ability to resist heat, NDV strains can be classified as either thermolabile or thermostable. The thermolabile strains lose their vitality after exposure at 50 to 55°C for about 30 min, while the thermostable strains still have hemagglutinating activity and infectivity for at least 30 min at 56°C (4–6).

The molecular basis for the thermostability of NDV has not been revealed completely, but previous studies have revealed some hints. In 1995, Tan et al. (7) compared the HN sequences of NDV strain AF2240, which is highly thermostable, with 13 other previously published sequences and found that the Arg (403) deletion in the HN gene might contribute to thermostability. In 2006, however, Kattenbelt et al. (8) did not find such a deletion in several heat-tolerant NDV isolates. Instead, they found that the majority of sequence differences between the I2 parental stock and the themostable I2 master seed virus stock were located in the L protein, speculating that the alternations in the L protein were responsible for the thermostable phenotype. In 2016, Wen et al. (9) generated chimeric viruses by exchanging viral genes between the thermostable TS09-C strain and the thermolabile LaSota strain using reverse-genetics technology. Evaluations of these chimeric NDV strains demonstrated that the thermostability of NDV was dependent on the origin of the HN protein.

NDV strain HR09 was isolated in China in the 1990s. We found that it has heat resistance and can remain viable at 56°C for at least 60 min. To investigate the strain’s phylogenetic relationship and molecular basis for heat resistance, we determined its complete genome sequence. The results show that it has a genome length of 15,192 nucleotides, consisting of six genes in the following order: 3′-NP-P-M-F-HN-L-5′. The genome has a G+C content of 46%. The acknowledged cleavage site of the F protein showed an amino acid sequence of 112-R-R-Q-K-R-L-117, which corresponds to those of virulent NDV strains. The BLASTn results showed that HR09 is most similar to strain AF2240 isolated from Malaysia, with 94% sequence identity. Phylogenetic analysis using the complete genome and complete coding sequence of the F gene according to the unified NDV classification system showed that HR09 belongs to genotype VIII and that it is related more closely to the velogenic strains QH1, QH4, and AF2240 than it is to other NDV strains. Characterization of the F and HN proteins of HR09 revealed a total of 2.7% to 10.5% amino acid substitutions compared with the thermostable vaccine strains V4, I-2, and TS09-C.

### Accession number(s).

The complete genome sequence of HR09 has been deposited in GenBank under the accession number MF285077.
